# Supplementary material for: Hyperuricemia and Its Association with Osteoporosis in a Large Asian Cohort
Source: Nutrients. 2022 May 26;14(11):2206. doi: 10.3390/nu14112206 (PMC9182899; doi:10.3390/nu14112206)
Supplement: Supplementary file 1 [file nutrients-14-02206-s001.zip › nutrients-1687751-supplementary.pdf]

Supplementary Figure S1. Directed acyclic graph showing the implicitly assumed causal relationship (between hyperuricemia and osteoporosis) underlying our adjusted models. We identified possible covariates through a literature search for common risk factors for osteoporosis and hyperuricemia. We also considered risk factors common to osteoporosis and hyperuricemia as possible covariates.

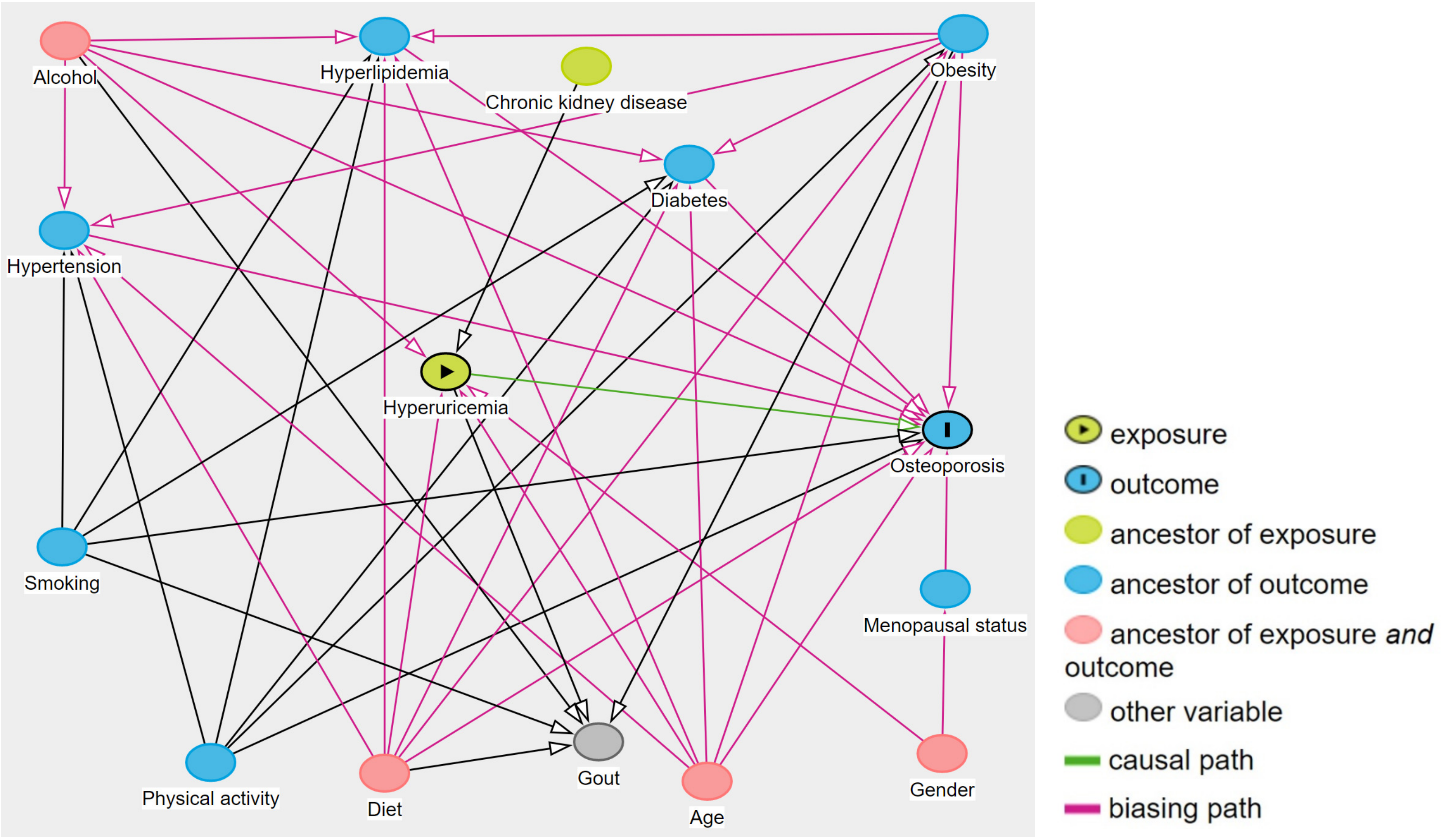

Supplementary Table S1. The association between serum uric acid (mg/dL) and estimated bone mineral density (g/cm^2) in all participants and in subgroup analyses by using a multivariable linear regression.

	**Adjusted β (95% CI)**	***p***
Serum uric acid	0.015 (0.009 to 0.025)	<0.001
Stratified by age and gender		
Gender, female, > 65 years old (N = 4,757)	0.072 (0.045 to 0.117)	0.020
Gender, female, ≦ 65 years old (N = 71,556)	0.015 (0.012 to 0.035)	<0.001
Gender, male, > 65 years old (N = 3,763)	0.010 (-0.025 to 0.044)	0.580
Gender, male, ≦ 65 years old (N = 38,961)	0.010 (-0.001 to 0.022)	0.064
Stratified by the presence of hyperuricemia		
Hyperuricemia (-), control group in the present study	0.020 (0.022 to 0.046)	<0.001
Hyperuricemia (+)	-0.017 (-0.050 to -0.003)	0.029
Stratified by the presence of diabetes mellitus		
Diabetes mellitus (-)	0.015 (0.009 to 0.025)	<0.001
Diabetes mellitus (+)	0.006 (-0.021 to 0.034)	0.647
Stratified by body mass index		
Body mass index < 30 kg/m2	0.011 (0.005 to 0.021)	0.002
Body mass index ≧ 30 kg/m2	0.019 (-0.003 to 0.046)	0.087

CI = Confidence interval.

Multivariable model: adjustment for age, gender, body mass index, smoke status, alcohol status, regular physical activity, presence of menopause, systolic blood pressure, diastolic blood pressure, history of hypertension, history of diabetes mellitus, history of dyslipidemia, history of gout, fasting glucose, cholesterol, triglyceride, low-density lipoprotein cholesterol, high-density lipoprotein cholesterol, serum albumin and creatinine.
